# Effect of the assignment of ancestral CpG state on the estimation of nucleotide substitution rates in mammals

**DOI:** 10.1186/1471-2148-8-265

**Published:** 2008-09-30

**Authors:** Daniel J Gaffney, Peter D Keightley

**Affiliations:** 1 McGill University and Genome Québec Innovation Centre, 740 ave Dr Penfield Rm 7208, Montréal (Québec), H3A 1A4, Canada; 2 Institute of Evolutionary Biology, School of Biological Sciences, University of Edinburgh, Edinburgh EH9 3JT, UK

## Abstract

**Background:**

Molecular evolutionary studies in mammals often estimate nucleotide substitution rates within and outside CpG dinucleotides separately. Frequently, in alignments of two sequences, the division of sites into CpG and non-CpG classes is based simply on the presence or absence of a CpG dinucleotide in either sequence, a procedure that we refer to as CpG/non-CpG assignment. Although it likely that this procedure is biased, it is generally assumed that the bias is negligible if species are very closely related.

**Results:**

Using simulations of DNA sequence evolution we show that assignment of the ancestral CpG state based on the simple presence/absence of the CpG dinucleotide can seriously bias estimates of the substitution rate, because many true non-CpG changes are misassigned as CpG. Paradoxically, this bias is most severe between closely related species, because a minimum of two substitutions are required to misassign a true ancestral CpG site as non-CpG whereas only a single substitution is required to misassign a true ancestral non-CpG site as CpG in a two branch tree. We also show that CpG misassignment bias differentially affects fourfold degenerate and noncoding sites due to differences in base composition such that fourfold degenerate sites can appear to be evolving more slowly than noncoding sites. We demonstrate that the effects predicted by our simulations occur in a real evolutionary setting by comparing substitution rates estimated from human-chimp coding and intronic sequence using CpG/non-CpG assignment with estimates derived from a method that is largely free from bias.

**Conclusion:**

Our study demonstrates that a common method of assigning sites into CpG and non CpG classes in pairwise alignments is seriously biased and recommends against the adoption of *ad hoc *methods of ancestral state assignment.

## Background

In mammals, the methylated form of cytosine (5-methylcytosine or 5mC) is hypermutable [1]. 5mC is formed by the enzyme DNA methyltransferase operating on a cytosine occurring immediately 5' of a guanine. One effect of methylation is to increase the rate of spontaneous deamination of 5mC to form thymine. It has been estimated that transitions in the methylated CpG dinucleotide occur 8–16 times faster than non-CpG transitions [2-4]. A smaller elevation of the rate of transversion mutation at the CpG dinucleotide has also been observed [3,5,6]. It has been suggested that CpG mutability in mammals underwent an abrupt elevation sometime around the mammalian radiation (~90 Myr; ref [2]), possibly in response to invasion by rapidly replicating transposable elements [7].

As a result of this hypermutability, molecular evolution studies have frequently attempted to estimate CpG and non-CpG substitution rates by separating observed substitutions into those that are inferred to have occurred within and outside CpG dinucleotides. In many previous studies [8-16], any nucleotide occurring within a CpG (i.e. either the constituent "C" or "G" of a CpG) or opposite a CpG (i.e. a site that, whilst not necessarily occurring within a CpG dinucleotide, is aligned with the "C" or "G" of a CpG dinucleotide in an orthologous sequence) has been inferred to have been an ancestral CpG site. We hereafter refer to this process as CpG/non-CpG assignment. Although CpG/non-CpG assignment is likely to be biased, it is generally assumed that this bias will be negligible if two sequences are closely related. Furthermore, some studies that employed this assignment procedure in the analysis of protein-coding sequence have suggested that while the overall rate of substitution is higher at synonymous sites, both CpG and non-CpG synonymous substitution rates are substantially lower than substitution rates in noncoding DNA [10,14]. This has been interpreted as evidence of purifying selection at synonymous sites.

**Figure 1:**
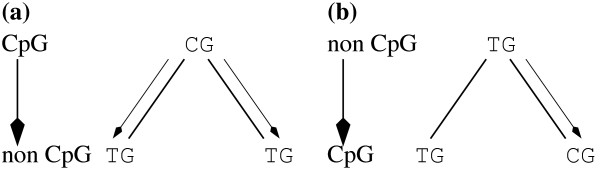
**Examples of CpG and non-CpG misassignment**. Arrows along lineages denote a single nucleotide substitution.

However, denoting a site as ancestrally CpG based on the presence of a CpG in one of two extant species is clearly biased: this method of ancestral state assignment implies that misclassification of an ancestral non-CpG as CpG in two lineages requires only a single change whereas a minimum of two changes, one in each lineage, must occur to misclassify an ancestral non-CpG as CpG (Figure 1). This is problematic because the probabilities of each misclassification will rarely equal each other and are conditional on the level of divergence of the two sequences in question. Furthermore, this bias may affect fourfold degenerate and noncoding sites differentially, since there are substantial compositional differences between synonymous and noncoding sites that arise, at least in part, because of the constraints imposed by the genetic code. The purpose of this study is to investigate the effects of misassignment of ancestral CpG state on the estimation of nucleotide substitution rates and how the level of misassignment varies with CpG hypermutability and evolutionary divergence. We deal explicitly with rates of nucleotide substitution between species.

## Results

### CpG/non-CpG assignment bias at mutational equilibrium

**Figure 2:**
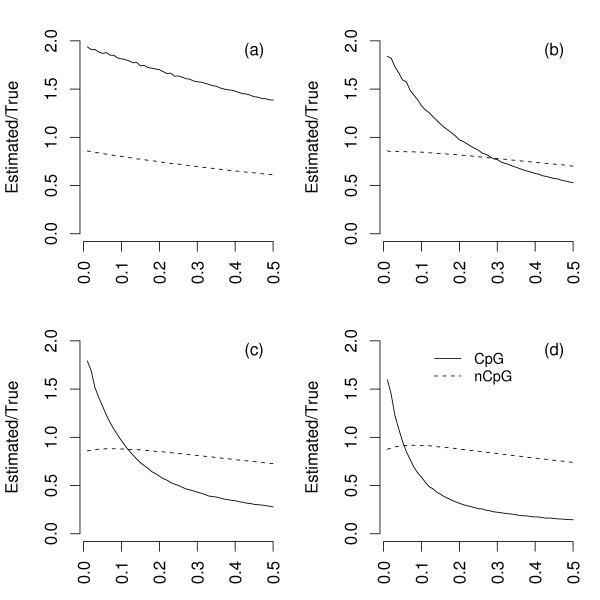
**Substitution rates at mutational equilibrium**. Ratio of estimated to true CpG differences and estimated to true non-CpG differences as a function of increasing sequence divergence for phylogenies derived from randomly generated sequences evolved to be at mutational equilibrium. Results are shown for four different levels of hypermutability: no hypermutability, 5-fold, 10-fold and 20-fold hypermutability (a, b, c and d). Each line represents 50 data points, each of which was estimated from the evolution of a single, randomly-generated 3 Mb sequence.

We first studied the simplest case of DNA sequences that had been evolved to approximate mutational equilibrium. DNA sequences were evolved to approximate mutational equilibrium by generating a random sequence and evolving until each site had experienced on average ten substitutions [17]. Two sequences derived from this ancestor were then evolved to a preset divergence. Pairwise divergence at CpG and non-CpG sites was then estimated using CpG/non-CpG assignment. It is clear that CpG/non-CpG assignment seriously compromises the accuracy of estimation of both the number of CpG and non-CpG changes, most notably in minimally-diverged sequences (Figure 2). Here, the number of true CpG changes is overestimated and the number of true non-CpG changes is underestimated. The explanation for this effect is as follows. For an ancestral CpG site to be assigned as non-CpG requires the destruction of the CpG site in both derived lineages, necessitating a minimum of two changes across the tree (Figure 1a). For an ancestral non-CpG site to be assigned as CpG requires only a single change (Figure 2b). The former will occur much less often (proportional to the square of the mean CpG divergence) in closely related species than the latter (which is linear in the mean divergence at non CpG sites). Thus many more ancestral non-CpGs will be misassigned as CpG in two closely related sequences than *vice versa*. However, the key point is that all true non-CpG dinucleotides that are misassigned will, by definition, have experienced at least a single change in one lineage. By incorrectly placing many true non-CpG changes in the CpG class we artificially inflate the estimate of the CpG rate, whilst depressing the estimate of the non-CpG rate. The difference in magnitude of the bias between CpG and non-CpG sites reflects the difference in total numbers of each site type.

### CpG/non-CpG assignment bias in coding and noncoding sequences

**Figure 3:**
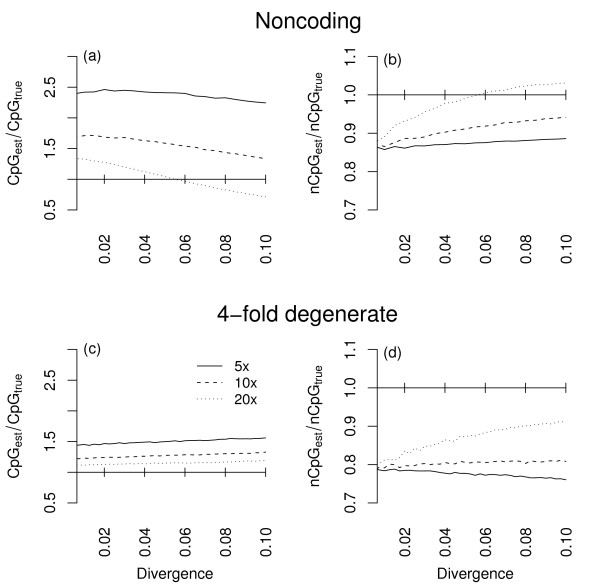
**Substitution rates in phylogenies derived from real sequence data**. Ratio of estimated (CpG_*est*_) to true (CpG_*true*_) CpG differences and estimated (nCpG_*est*_) to true (nCpG_*true*_) non-CpG differences plotted against increasing sequence divergence for phylogenies derived from real mouse noncoding (a, b) and coding (c, d) sequences. Separate lines show results for different levels of hypermutability (5-fold, 10-fold and 20-fold hypermutability). Each line is composed of is estimated from 80 simulated replicates of an ~8 Mb sequence.

We next investigated the impact of CpG/non-CpG misassignment in sequence data that were compositionally more realistic than randomly generated sequences at approximate mutational equilibrium. Real mouse coding and intronic sequences were used as ancestral sequences and again, two derived sequences were copied, evolved and the number of CpG and non-CpG changes estimated using CpG/non-CpG assignment. The results of this analysis are presented in Figure 3. As observed at approximate mutational equilibrium (Figure 2) the number of CpG substitutions tends to be overestimated and number of non-CpG substitutions tends to be underestimated at both fourfold and noncoding sites. Interestingly, however, the magnitude of overestimation of the number of CpG changes tends to be larger in noncoding DNA. For example, at 10-fold hypermutability and a pairwise divergence of 1%, the number of CpG substitutions in noncoding DNA is overestimated by ~50%, whereas the equivalent figure is ~17% at fourfold sites. Similarly, the magnitude of underestimation of non-CpG changes is lower at noncoding sites. For the same parameter combination as described above, the number of non-CpG substitutions is underestimated by ~12% in noncoding DNA, and by ~20% at fourfold sites.

The difference in level of estimation bias at fourfold and noncoding sites results from a difference in CpG content. Because of the structure of the genetic code, fourfold degenerate sites are typically enriched for CpG dinucleotides, whereas noncoding sequences are depleted (0.032 vs 0.010 in our murid sequences). With changing CpG frequency, the numbers of misassigned sites will make up different proportions of the total lost or gained from the CpG and non-CpG classes. Thus with decreasing CpG content, the overestimation of the CpG substitution rate becomes greater whereas the underestimation of the non-CpG substitution rate becomes less. Paradoxically, this result implies that whenever CpG/non-CpG assignment is used to estimate substitution rates between closely related species, CpG-rich sequences will appear to be evolving more slowly than CpG-poor sequences.

### CpG/non-CpG assignment bias in real sequence data

We next investigated whether the effects predicted by our simulations could be observed in a real evolutionary setting. As the CpG/non-CpG assignment bias primarily affects closely related sequences, we estimated substitution rates in human and chimpanzee pairwise alignments of coding and noncoding sequence. As we cannot know the true numbers of CpG and non-CpG substitutions in a real alignment, we sought to compare CpG/non-CpG assignment with a bias-free method. Previous studies [18-20] have employed a "preC", "postG" classification in order to remove CpG mutations from nucleotide sequence data. Further simulations showed that excluding all sites preceded by C and followed by G ("CpG prone sites"), reliably removes the effects of CpG mutation [see Additional File 1] although this removes a substantial number of sites from our dataset (~65% of all fourfold degenerate sites and ~%36 of all intronic sites are removed). We compared the non-CpG prone substitution rate with the substitution rate estimated at non-CpG sites identified using CpG/non-CpG assignment. The results of this analysis are presented in Table 1. As predicted by our simulations, the substitution rate at non-CpG sites, identified using CpG/non-CpG assignment, is underestimated (by 23% and 13% at fourfold and intronic sites, respectively) when compared with the bias-free method. The difference between substitution rates at non-CpG and non-CpG prone sites is highly significant both in introns and at fourfold sites (P < 0.0001; bootstrap test). The ratio of synonymous to intronic substitution rates is also significantly lower (P < 0.0001; bootstrap test) at non-CpG sites compared with non-CpG prone sites. As in our simulations, this likely results from differences in CpG content between fourfold and intronic sites, that produce different degrees of underestimation of the non-CpG rate when CpG/non-CpG assignment is used. Thus, CpG/non-CpG assignment could be potentially misleading when comparing CpG rich and CpG poor sequences, such as synonymous and intronic sites.

**Table 1:** Nucleotide substitution rates estimated at non-CpG (*K*_*nCG*_) and non-CpG prone (*K*_*nCGprone*_) sites at fourfold degenerate and intronic sites in 1470 human-chimpanzee orthologues.

	*K*_*nCG*_	*K*_*nCGprone*_	*K*_*nCG*_/*K*_*nCGprone*_
4-fold	0.0061 (0.0058,0.0064)	0.0079 (0.0073,0.0084)	0.771
Intronic	0.0092 (0.0089,0.0095)	0.0105 (0.0102,0.0109)	0.876
			
*K*_4_/*K*_*Int*_	0.663 (0.621,0.704)	0.750 (0.692,0.810)	

Non CpG sites were identified using CpG/non-CpG assignment. Numbers in parentheses show 95% confidence intervals estimated by bootstrapping the data by gene, 10000 times.

## Discussion

Reliable, bias-free estimation of nucleotide substitution rates is a fundamental part of molecular evolutionary studies. Furthermore in order to test hypotheses about natural selection, underlying neutral processes, such as mutational rate variation, must be accounted for and their effects quantified or removed as efficiently as possible. Our study has shown that a commonly used method of effecting such a removal, assigning nucleotide sites to CpG and non-CpG categories, systematically biases the estimation of nucleotide substitution rates. In particular, across small to moderate evolutionary distances, CpG/non-CpG assignment seriously upwardly biases the estimate of the number of CpG changes and downwardly biases the estimate of the number of non-CpG changes. This is due to a simple artefact that causes many more true non-CpG changes to be misassigned as CpG than *vice versa*. Clearly, more reliable removal of the effects of CpG mutation is required prior to any meaningful comparison of rates of nucleotide substitution among different regions of mammalian genomes. Our simulations indicated that exclusion of non-CpG-prone sites (sites preceded by "C" and/or followed by "G"), is one simple and effective method of removing CpG-derived changes. One possible alternative to this is to employ an outgroup species to improve the accuracy of ancestral assignment. However simulations showed that parsimony-based ancestral assignment can introduce additional biases into the estimation of substitution rates [see Additional File 1]. These effects are consistent with the known problems with parsimony when base composition is biased [21], namely an excess of common-to-rare changes (in this case an excess of non CpG to CpG changes).

The bias we describe here also causes the substitution rate within CpG dinucleotides to be substantially overestimated between closely related species. Estimation of the rate of substitution at CpGs has also been attempted using CpG/non-CpG assignment by previous studies [8,10]. It is likely that such studies will have overestimated the rate of substitution at CpG sites, again due to misassignment issues. When estimating the rate of substitution at CpG sites, it is clearly preferable to implement a method that explicitly models context-dependent evolution, such those proposed in refs [3,4,22] or [23]. Our results are supported by recent studies that have demonstrated that methods of "optimal" ancestral state assignment, such as the *ad hoc *method described here, can be seriously biased and misleading [24,25].

The results presented here also have implications for previous comparisons of the substitution rate at fourfold and noncoding sites in mammalian genomes [10,14]. As a result of CpG/non-CpG misassignment bias and differences in base composition, typical mammalian noncoding and fourfold degenerate sites are likely to be predisposed to misleading inferences about their relative rates of substitution. The net effect is that fourfold synonymous sites appear to be evolving more slowly than noncoding sites when substitutions are divided into those that have apparently occurred within and outside a CpG dinucleotide. Furthermore, fourfold sites will typically have a higher CpG frequency than noncoding sites both during the progression to, and at, mutational equilibrium. Because of this, an apparent depression of evolutionary rates at fourfold degenerate sites, when compared to noncoding DNA using CpG/non-CpG assignment, is likely to be a general feature of mammalian molecular evolution.

## Conclusion

Our study shows that a commonly used method of assigning sites to CpG or non CpG classes in pairwise alignments is seriously biased. We further note that the effects of CpG and non-CpG misassignment at fourfold and noncoding sites are dependent on differential CpG frequencies, and so these results will apply to any comparison of substitution rates where this is the case. Our results therefore recommend against the adoption of *ad hoc *methods of ancestral state assignment.

## Methods

We studied a simple mutation model in which transitions occurred twice as frequently as transversions, and CpG mutations occurred at a different frequency to non-CpG mutations. In all our simulations, two sequences were copied from a single ancestral sequence and evolved. In molecular evolutionary studies fourfold degenerate sites in codons that code for different amino acids in both derived sequences are excluded. To simplify this, in our simulated coding sequences, all nonsynonymous substitutions were considered strongly deleterious and rejected. Qualitatively similar results were obtained when a given proportion of nonsynonymous changes were modelled as neutral (results not shown). In all cases, estimates of the numbers and rates of nucleotide substitution were corrected for multiple hits using the Jukes-Cantor model [26]. This was to ensure simplicity in the interpretation of our results given that more parameter-rich multiple hits corrections take base composition into account, and it is unclear whether this is appropriate when dividing sites into CpG and non CpG. Our data were not simulated under a JC model, since we included a variable transition/transversion rate. However, varying the transition/transversion did not qualitatively impact our results (data not shown).

Ancestral sequences in our simulations were derived from two sources: randomly generated sequences that were evolved to reach approximate mutational equilibrium, and real sequences derived from the mouse genome. The former allowed us to quantify the effects of CpG misassignment bias free of the complicating effects of nonequilibrium processes. Real mouse coding and intronic sequences (collected in ref [27]) were used in order to capture the base composition and nonequilibrium evolution characteristic of mammalian genomes in our simulations [17,28-30]. To investigate the level of bias in a real evolutionary scenario we also collected a random sample of 2000 human RefSeq genes for which we extracted orthologous chimpanzee sequence from the UCSC whole genome alignments. All alignments that did not start with ATG, included premature stop codons or in which the sequence length was not a multiple of three in either species were removed leaving a total of 1470 genes. We removed CpG islands from our intronic sequences using the criteria of [31].

## Authors' contributions

DJG and PK designed research. DJG performed research and wrote the paper.

## Supplementary Material

Additional file 1 Supplementary Figures. Figure 1 shows the effect of removing non CpG-prone sites on removing the influence of CpG derived mutations. Figure 2 shows the effect of the inclusion of information from a moderately diverged outgroup species on the inference of ancestral CpG status. Click here for file
