# Supplementary material for: Effect of the assignment of ancestral CpG state on the estimation of nucleotide substitution rates in mammals
Source: BMC Evol Biol. 2008 Sep 30;8:265. doi: 10.1186/1471-2148-8-265 (PMC2576242; doi:10.1186/1471-2148-8-265)
Supplement: Additional file 1 — Supplementary Figures. Figure 1 shows the effect of removing non CpG-prone sites on removing the influence of CpG derived mutations. Figure 2 shows the effect of the inclusion of information from a moderately diverged outgroup species on the inference of ancestral CpG status. [file 1471-2148-8-265-S1.pdf]

Supplementary Figures

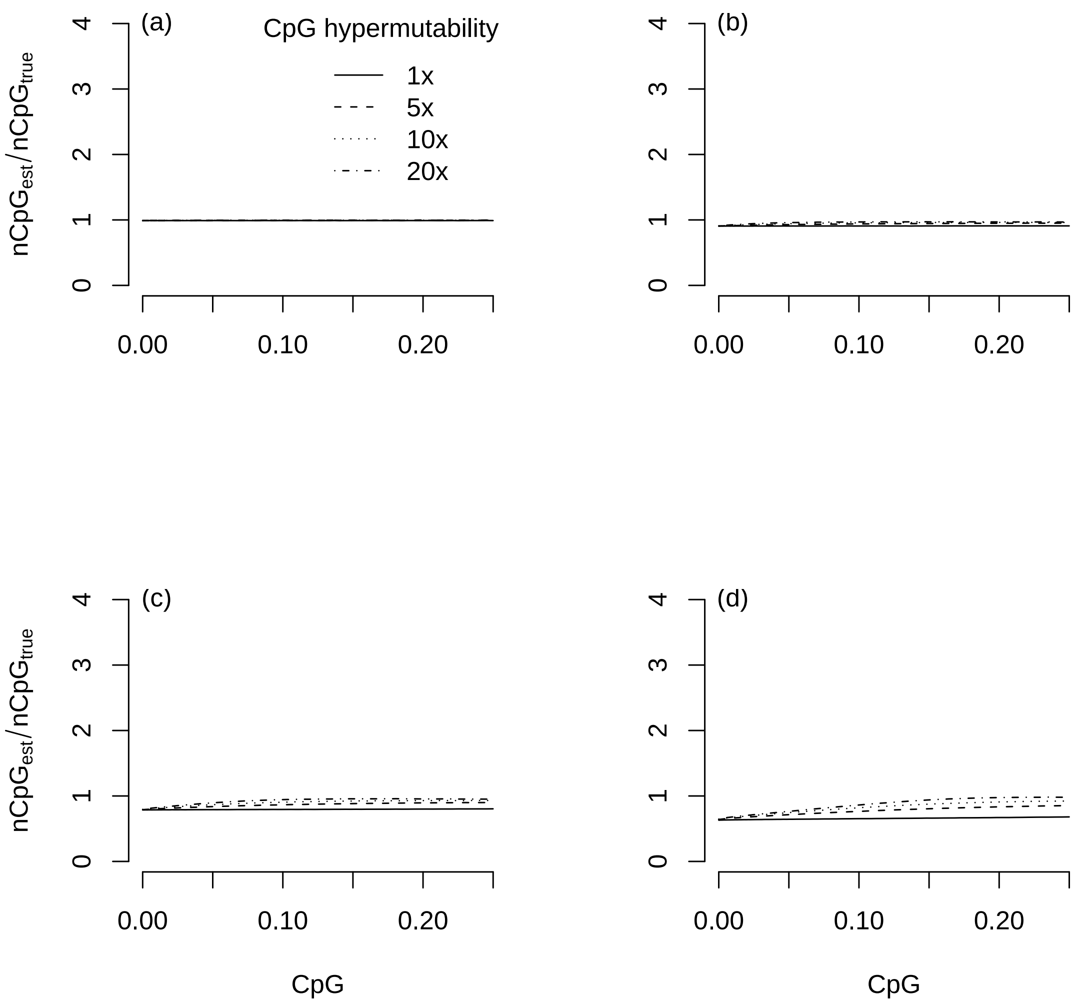

Figure 1: Ratio of estimated (nCpGp$_{est}$) to true (nCpGp$_{true}$) non CpG-prone differences across a variety of ancestral CpG frequencies, in phylogenies derived from "artificial ancestral" sequences. Results are from trees of total length 0.01, 0.05, 0.1 and 0.5 (a,b,c and d respectively). Separate lines show results for different levels of hypermutability (no hypermutability, 5-fold, 10-fold and 20-fold hypermutability). Each line represents 101 data points, each of which represents 10 simulated replicates of a 1Mb sequence.

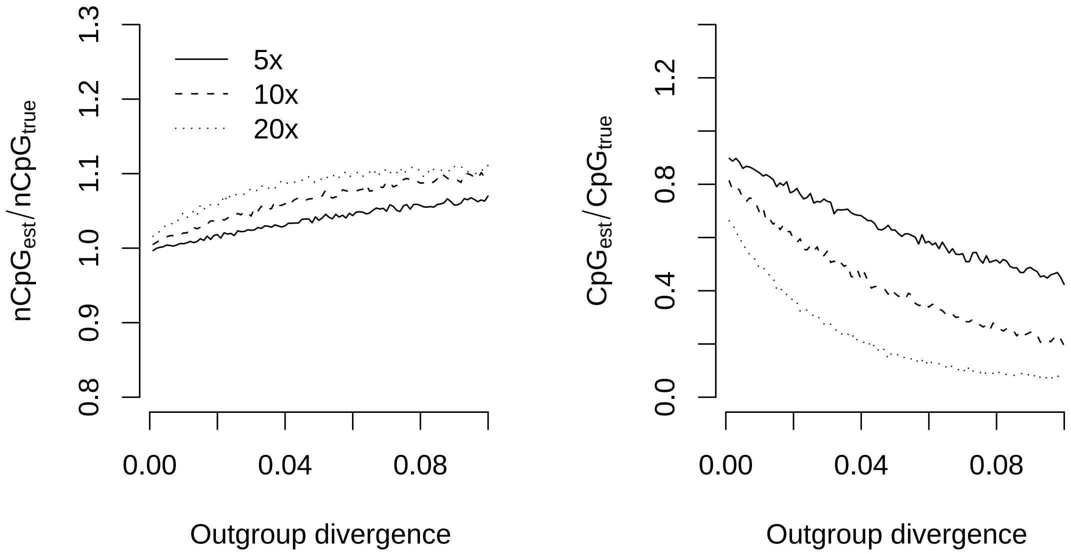

Figure 2: Ratio of estimated to true CpG differences and estimated to true non-CpG differences as a function of increasing outgroup sequence divergence for three-branch phylogenies derived from randomly generated sequences evolved to be at mutational equilibrium. Estimated numbers of CpG and non-CpG changes are corrected for multiple hits using the Jukes-Cantor model. Results are shown for three different levels of hypermutability: 5-fold,10-fold and 20-fold hypermutability. Each line represents 50 data points, each of which was estimated from the evolution of a single, randomly-generated 1Mb sequence.
